# m^6^A-Related Angiogenic Genes to Construct Prognostic Signature, Reveal Immune and Oxidative Stress Landscape, and Screen Drugs in Hepatocellular Carcinoma

**DOI:** 10.1155/2022/8301888

**Published:** 2022-09-30

**Authors:** Xiaodong Qu, Luyao Zhang, Songbo Li, Tian Li, Xingyu Zhao, Na Wang, Yongquan Shi

**Affiliations:** ^1^State Key Laboratory of Cancer Biology, National Clinical Research Center for Digestive Diseases, Xijing Hospital, Fourth Military Medical University, Xi'an 710032, China; ^2^School of Basic Medicine, Fourth Military Medical University, 169 Changle West Rd, Xi'an 710032, China

## Abstract

**Background:**

m^6^A modification plays a key role in the development of hepatocellular carcinoma (HCC). Angiogenesis-related genes (ARGs) are increasingly being used to define signatures predicting patient prognosis. The correlations between m^6^A-related ARGs (mARGs), clinical outcomes, and the immune and oxidative stress landscape are unclear.

**Methods:**

Univariate Cox regression analysis of 24 mARGs yielded 13 prognostic genes, which were then analyzed for their enriched functions and pathways. After LASSO regression analysis, a prognostic signature was constructed and its reliability validated. Patients were grouped by risk using the signature score, and then the clinical prognosis, the immune landscape, and the oxidative stress landscape between the two groups were analyzed. Drug sensitivity analysis was performed to identify potentially efficient therapeutic agents.

**Results:**

Thirteen prognosis-related mARGs consistently clustered patients with HCC into four groups with significantly different prognosis. Four mARGs (*EGF*, *ITGA5*, *ITGAV*, and *PLG*) were used to construct a prognostic signature and define risk groups. Among them, *EGF*, *ITGA5*, and *ITGAV*, were defined as prognostic risk factors, while *PLG* was defined as a prognostic protective factor. Compared to low-risk patients, HCC patients in the high-risk group had a poorer prognosis and showed significant differences in clinical characteristics, enriched pathways, tumor stemness, and tumor microenvironment. The drug sensitivity of oxaliplatin and LDK-378 negatively correlated with ITGAV expression. Ten drugs had lower IC50s in the high-risk group, indicating better antitumor efficacy than in the low-risk group, with epothilone B having the lowest IC50 value.

**Conclusions:**

A prognostic model consisting of mARGs can be used to predict the prognosis of HCC patients. The risk grouping of our model can be used to reveal differences in the tumor immune microenvironment of patients with HCC. Further in-depth study may provide new targets for future treatment.

## 1. Introduction

Primary liver cancer, with approximately 906,000 new cases and 830,000 deaths in 2020, has become the sixth most common malignancy in terms of incidence, placing a heavy burden on individuals and healthcare systems worldwide [1]. Hepatocellular carcinoma (HCC) represents 75% of primary liver cancers [2]. With the growing evolution and amelioration in therapeutic strategies [3], the survival rate of HCC patients has improved [4]. However, HCC is prone to metastasis and recurrence, with 40–70% of patients experiencing recurrence after liver resection [5]. The 5-year survival rate is less than 20%, which seriously affects the therapeutic outcome and the quality of life of the survivors [6]. Given the complex pathogenesis of HCC and the general unpromising prognosis, it is urgently necessary to build an effective signature to forecast the prognosis and to discover new potential therapeutic agents.

N6-methyldosine (m^6^A) modification occurs in approximately one-fourth of mRNA sequences and is one of the most widely used RNA methylation modifications in mammals [7, 8]. m^6^A modifications can participate in the regulation of RNA metabolism by participating in translational regulation, noncoding RNA processing, and mRNA decay [9–11]. m^6^A regulators can be divided into three categories according to their functions: writers, erasers, and readers, which participate in HCC through multiple mechanisms [12]. ALKBH5-mediated IGF2BP1-dependent m^6^A demethylation reduces the stability of LYPD1 mRNA, and aberrant downregulation of ALKBH5 promotes LYPD1 expression and induces oncogenic behavior of HCC [13]. METTL14 also promotes the stability of HNF3*γ* mRNA through IGF2BPs-dependent m^6^A modification, resulting in the upregulation of HNF3*γ* expression, which has been shown to play an important role in HCC stem cells differentiation and in regulating the sensitivity of HCC cells to sorafenib treatment [14]. Under hypoxic conditions in HCC, upregulated YTHDF1 contributes to the translation of autophagy-related genes ATG2A and ATG14, thereby promoting autophagy and autophagy-associated malignancy [15]. WTAP-mediated m^6^A modification leads to posttranscriptional repression of ETS1, and WTAP can inhibit ETS1 expression by interfering with the binding of HuR protein to ETS1 mRNA [16]. The m^6^A modification is also extensively involved in the regulation of noncoding RNAs. METTL3 increases the stability of Linc00958 transcript and upregulates its expression. Subsequently, Linc00958 regulated miR-3619-5p/HDGF axis to promote HCC progression [17]. Similarly, IGF2BP1 and METTL3/14 promote the stability of circMDK and circRNA-SORE, respectively, to upregulate their expression and thus play a role in the development of HCC [18, 19]. Unlike most circular RNAs, circMAP3K4 was found to have coding potential, and IGF2BP1 recognizes circMAP3K4 N6-methyladenosine modification and promotes its translation, thereby protecting HCC cells from cisplatin exposure [20]. Furthermore, bioinformatics analysis suggests that m^6^A regulators may be closely related to the prognosis of HCC patients [21].

Angiogenesis, which provides sufficient oxygen and nutrients for tumors, is the basis for tumor growth. In tumor tissue, after local degeneration of the basement membrane around the capillaries, endothelial cells persistently invade to form three-dimensional scaffolds that connect with other similar structures to develop new blood vessels [22]. Angiogenesis in tumor tissue is regulated by several growth factors and chemokine signals [23]. Specific knockdown of VEGF in HCC has been reported to inhibit endothelial cell function and suppress angiogenesis [24]. Furthermore, VEGF can function as a prognosis, recurrence, and treatment response predictor for HCC [25]. Since angiogenesis serves important functions in the development of HCC, abnormal expression of angiogenesis-related genes (ARGs) may be helpful in anticipating the prognosis of HCC patients.

In many previous studies, a close link between m^6^A and angiogenic processes has been revealed. For example, Qiao et al. [26] found that the m^6^A writer, METTL3, was closely associated with vasculogenic mimicry (VM) in HCC tissues. They further demonstrated that METTL3 could promote VM formation and malignant progression by improving the translation efficiency of YAP1 mRNA. Zhao et al. found that loss of ALKBH5 promotes postischemic angiogenesis by regulating the posttranscriptional stabilization of WNT5A in an m^6^A-dependent manner [27]. In a variety of diseases, m^6^A regulation and angiogenic processes are closely related to oxidative stress. The regulation of angiogenic processes by oxidative stress through VEGF-dependent and non-VEGF-dependent signaling pathways plays an important role in a variety of chronic diseases and tumors [28, 29]. Many studies have determined that oxidative stress during cancer may regulate m^6^A methylation through the accumulation of reactive oxygen species, while at the same time, the regulation of m^6^A can affect the biological function of cancer cells by influencing the levels of oxidative stress [30, 31]. Numerous HCC-related prognostic signatures have previously been constructed [32]. However, studies related to m^6^A-related angiogenesis in HCC are lacking, and no relevant prognostic signature has been developed that effectively predicts patient prognosis and reveals the landscape of oxidative stress in patients.

Therefore, our objective was to identify the m^6^A-related ARGs (mARGs) in HCC and to construct a validated prognostic signature. The prognostic signature was then applied to classify HCC patients according to the risk scores. We performed a signature-related prognosis, gene set enrichment analysis (GSEA), tumor microenvironment (TME), immune checkpoint gene, and clinical correlation analysis, and we also evaluated potential oncologic therapies.

## 2. Materials and Methods

### 2.1. Data Download and Processing

The mRNA sequencing information, clinicopathological characteristics data, and HCC genetic mutations were acquired from the Cancer Genome Atlas (TCGA) database [33], which consisted of 374 HCC samples and 50 adjacent normal samples. These 424 HCC samples were analyzed for differences in ARGs and m^6^A coexpression, of which 364 samples containing survival information were used for signature construction. The samples were randomly grouped, with 183 HCC samples as the training set and the remainder of the cases as the test set. The DNA copy number variations (CNV) data from these patients was obtained from the UCSC Xena website [34].

### 2.2. Identification of mARGs

We first searched the GeneCards Suite website [35] using the keyword “angiogenesis” to obtain genes encoding protein related to angiogenesis and obtained the scores calculated by the website for the correlation between genes and angiogenesis. We selected ARGs with a score>5 for subsequent analysis. We obtained differentially expressed ARGs using the “limma” package, and the statistical significance was set to |logFC| ≥1 and *P* < 0.05. The differential expression heat map was constructed using the “pheatmap” package. After obtaining data regarding the expression of m^6^A regulator genes (including m^6^A writers: *METTL3*, *METTL14*, *METTL16*, *WTAP*, *VIRMA*, *ZC3H13*, *RBM15*, *RBM15B*; m^6^A readers: *YTHDC1*, *YTHDC2*, *YTHDF1*, *YTHDF2*, *YTHDF3*, *HNRNPC*, *FMR1*, *LRPPRC*, *HNRNPA2B1*, *IGF2BP1*, *IGF2BP2*, *IGF2BP3*, *RBMX*; and m^6^A erasers: *FT0* and *ALKBH5*) through the package “limma,” we conducted a coexpression analysis of ARGs and 23 regulator genes of m^6^A by performing the “Wilcox test,” and |cor| >0.3 and *P* < 0.05 values were defined a correlation between genes. In addition, the coexpression network was visualized using “dplyr,” “ggalluvial,” “ggplot2,” and “igraph.” Protein–protein interaction (PPI) analysis was performed on the String website [36] to uncover interactions between mARGs. The screened mARGs were then used for signature construction.

### 2.3. Acquisition of Prognosis-Related mARGs and Consensus Clustering

After obtaining the expression of mARGs and survival information of the samples, the “survival” package was used to perform a univariate Cox regression analysis and obtain the prognosis-related mARGs and their corresponding coefficients. The packages “limma” and “ConsensusClusterPlus” were used to read gene expression data and consensus cluster analysis to select the optimal *k*-value to divide the sample into several clusters, and the heat map showed variations in clinical information and gene expression between these clusters.

### 2.4. Analysis of Prognosis-Related mARG Enrichment

To gain added insight into prognosis-related mARGs, the R software and the “RCircos” package were used to reveal the CNV of prognosis-related mARGs and to illustrate the location of prognosis-related mARGs on the chromosome. The “maftools” package was used to indicate the mutations of prognosis-related mARGs in HCC samples. The packages “clusterProfiler,” “(http://org.Hs.eg/).db,” “ggplot2,” “enrichplot,” and “GOplot” were used for Gene Ontology (GO) and Kyoto Encyclopedia of Genes and Genomes (KEGG) pathway enrichment analysis.

### 2.5. Construction of the Prognostic Signature and Subsequent Validation

Several prognostic mARGs were obtained from univariate Cox analysis, and then the packages “survival,” “caret,” “glmnet,” “survminer,” and “timeROC” were used to perform LASSO regression analysis in the training set to further filter signature genes. Based on the expression and coefficient values of the signature genes, the risk score of each sample was obtained using the following formula:

(1) $$\begin{array}{c} \text{Risk score}=\overset{n}{\underset{i=1}{\sum }}\left(\text{Coefficient}\left(\text{i}\right)*\text{Expr}\left(\text{i}\right)\right). \end{array}$$

The median risk score of the training set is served as the cutoff point for the risk subgrouping of the training set, the test set, and the entire set. The “survival” and “survminer” packages were used for the survival analysis of training, test, and entire sets. We separated the samples containing clinical information into the training set, and the test set only in the survival analysis, and in other analyses related to the gene signatures, the whole sample was also evaluated. In addition, the packages “rms,” “regplot,” “timeROC,” and “survival” were used to develop a nomogram. Our nomogram performance was evaluated using ROC curves.

### 2.6. Signature-Related Gene Set Enrichment Analysis, Cancer Stemness Analysis, and Genetic Mutation Status

Gene set enrichment analysis (GSEA) was performed using GSEA software (in version 4.0.3) to identify enriched KEGG signaling pathways in high- and low-risk groups based on all gene expression profiles and the “c2.cp.kegg.v7.5.symbols” gene set database. The count of permutations was set to 1000. Significantly enriched pathways were defined as those with *P* < 0.05 or false discovery rates (FDR) <0.25. The packages “limma,” “ggplot2,” “ggpubr,” and “ggExtra” were used to perform a correlation analysis between risk scores and HCC cell stemness. We use the “maftools” package to obtain mutation waterfall maps for the two risk groups after obtaining the mutation data from the HCC samples.

### 2.7. Signature-Related Immune Landscape Analyses

To confirm the potential of our signature in revealing TME, we used the “limma” package, the CIBERSORT [37] algorithm, which can calculate the relative content of 22 cell types by analyzing the gene expression data of the samples, and the “vioplot” package to visualize the variation in immune cell abundance between the two risk groups. Differences in immune-related pathways between the two risk groups were analyzed using the packages “limma,” “GSVA,” and “GSEABase.” The immune score, the stromal score, and the estimate score were calculated for each sample using the package “estimate” [38]. And the packages “reshape2,” “ggplot2,” “ggpubr,” and “ggExtra” were used to reveal differences in the immune score and stromal score between the high- and low-risk groups and the relationship between risk score, immune score, and the stromal score. The packages “limma,” “reshape2,” “ggpubr,” “ggplot2,” and “corrplot” were used to show the expression of immune checkpoint genes in the two risk groups and their correlation with signature genes.

### 2.8. Drug Screening and Drug Correlation Analysis

We downloaded drug experimental data of human cells from the CellMiner website [39] and used the “limma” and “impute” packages to perform sensitivity analysis between signature genes and drugs. In addition, gene expression profiles of HCC samples, information on risk grouping, and “pRRophitic” package [40] were used to predict semi-inhibitory concentrations (IC50) of drugs based on the drug list included in the Genomics of Drug Sensitivity in Cancer (GDSC) database [41]. Lower IC50 meant that the drug is more effective in treating cancer.

### 2.9. Collection of Patient Tissue Specimens and Quantitative Real-Time Polymerase Chain Reaction (qRT-PCR)

Fifteen pairs of human primary tumor tissue and normal tissue were obtained from patients who had not undergone radiotherapy prior to surgical resection. All patients had signed an informed consent form prior to providing specimens. Our study was approved by the Human Subjects Committee of the Xijing Hospital. After excision, fresh tissues were transferred simultaneously to liquid nitrogen for subsequent RNA extraction. Total RNA from human tissues was extracted using TRIzol (Invitrogen). The RNA was then reverse transcribed into cDNA using the PrimeScript RT kit (Takara), and qPCR was performed using SYBR Premix Ex Taq II (Takara) for real-time PCR detection (Bio-Rad). Supplementary Table 1 lists all primers used in PCR. The expression levels of the four signature genes were normalized to those of GADPH.

### 2.10. Statistical Analysis

R software (in version 4.0.3) was used to perform statistical analysis. *P* values <0.05 indicated differences were considered statistically significant.

## 3. Results

### 3.1. Acquisition and General Landscape of mARGs

**Figure 1:**
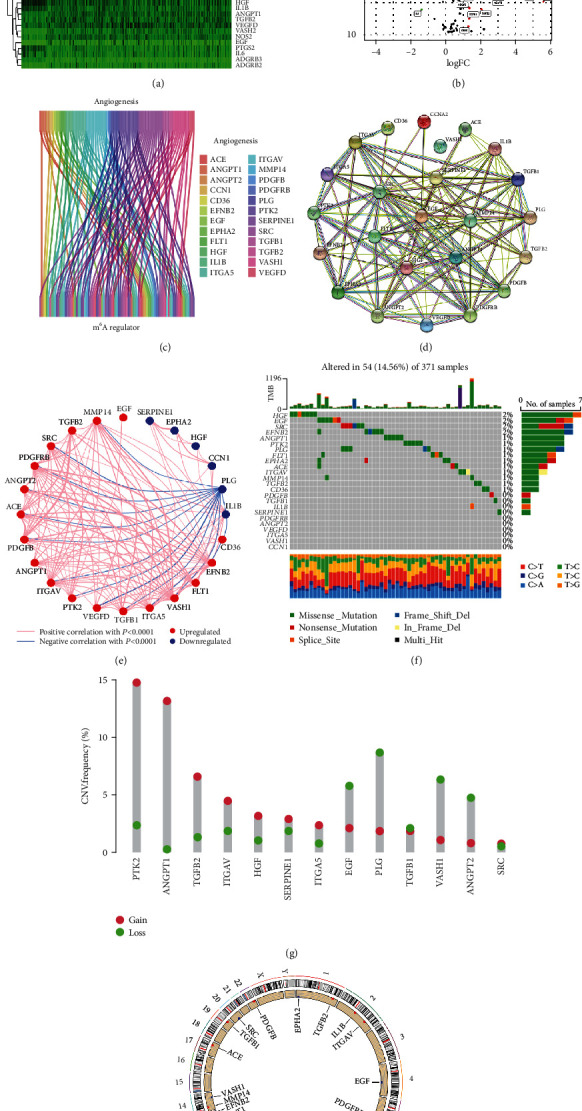
Acquisition of mARGs acquisition and genetic mutational landscape. (a and b) Heat map and volcano plot show differentially expressed ARGs. “N” stands for normal samples and “T” stands for HCC tumor samples. Red dots indicate upregulation of gene expression, green dots indicate downregulation of gene expression, and black dots indicate no significant change in gene expression. (c) The 24 mARGs screened by coexpression analysis between ARGs and m^6^A regulator gene. (d) The PPI network among the mARGs. (e) The network of mARGs correlations. The red line and the blue line indicate positive and negative correlations in gene expression, respectively. The red and blue dots indicate up- and downregulation of gene expression, respectively (*P* < 0.0001). (f) Mutation landscape of mARGs. (g) The CNV gain and loss frequencies among mARGs. (h) Distribution of the mARGs location on chromosomes.

The flow diagram of our study is illustrated in Supplementary Figure S1. Overall, 108 ARGs encoding proteins with score>5 were obtained from the GeneCards Suite website. Of these, 45 ARGs were differentially expressed in HCC and normal tissues (Figures 1(a) and 1(b), Supplementary Table 2). Then, 24 mARGs were obtained after coexpression analysis with m^6^A regulator gene (Figure 1(c), Supplementary Table 3).  Figure 1(d) illustrates the PPI analysis results of 24 mARGs through the STRING website tool to reveal the interactions between mARGs, and Figure 1(e) illustrates the constructed network of coexpression relationships. We revealed genetic mutations in 24 mARGs in HCC (Figure 1(f)). Fifty-four of the 371 HCC samples (14.56%) showed mutations in mARG, with *HGF* being the most mutated gene among the 24 mARG, followed by *EGF*, *SRC*, and *EFNB2*. In addition, Figures 1(g) and 1(h) show the CNV alterations as well as the altered loci on the chromosomes of the 24 mARGs. As the copy number of the 24 mARGs changed significantly, we assumed that CNV had a regulatory role in the expression of mARGs.

### 3.2. Screening and Analyzing Prognosis-Related mARGs in HCC

**Figure 2:**
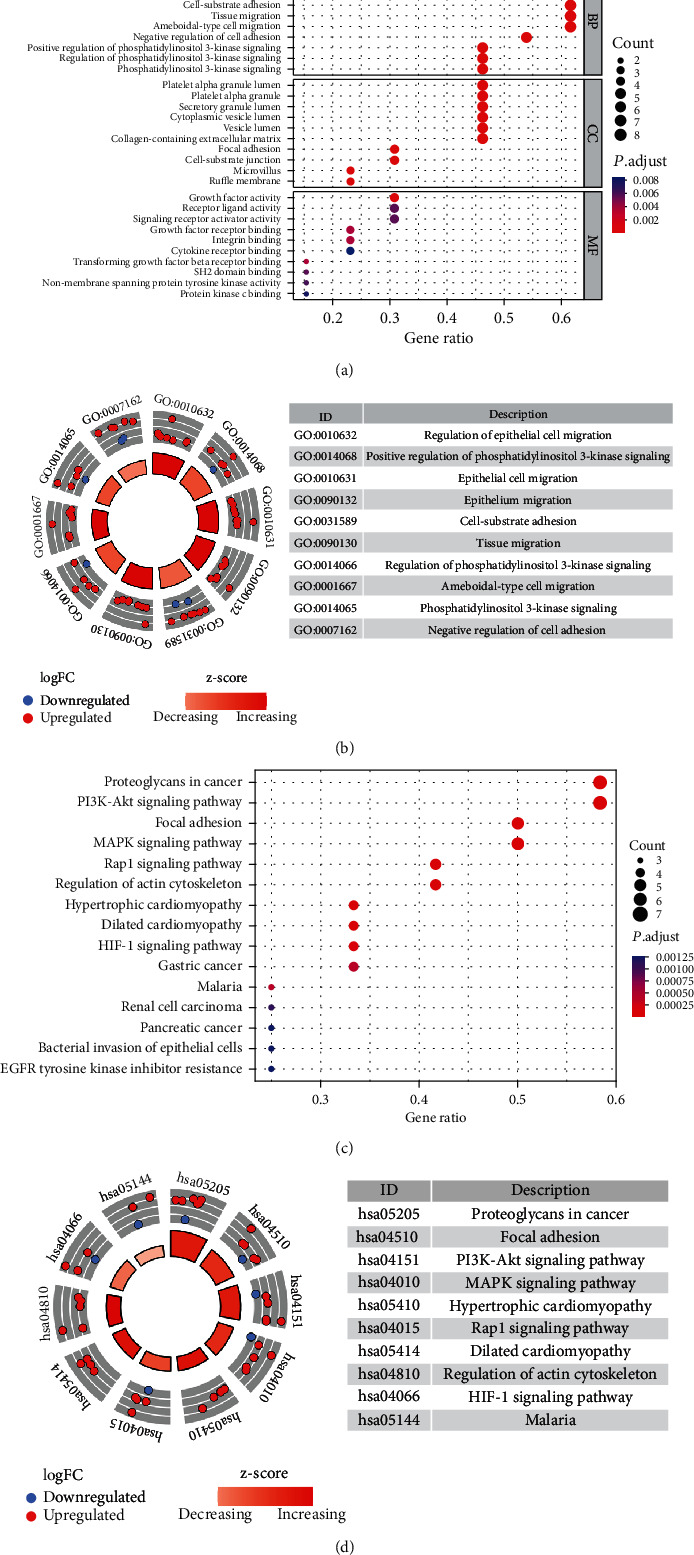
Functional and pathway analyses of prognosis-related mARGs. Bubble plot (a) and circle plot (b) of GO analysis of prognosis-related mARGs. Bubble plot (c) and circle plot (d) of KEGG analysis of prognosis-related mARGs.

We excluded samples without clinical prognostic data and conducted a univariate Cox analysis of the 24 mARGs with prognosis, identifying 13 prognostic mARGs (Table 1). The results of GO analysis are illustrated in Figures 2(a) and 2(b), and the biological process (BP) analysis revealed the regulation of epithelial cell migration, epithelial cell migration, cell-substrate adhesion, and epithelium migration. Cellular component (CC) analysis mainly included the lumen of the platelet alpha granule, the lumen of the platelet alpha granule, the secretory granule, and the lumen of the cytoplasmic vesicle lumen. Molecular function (MF) analysis revealed growth factor activity, receptor ligand activity, signaling receptor activator activity, and growth factor receptor binding. Furthermore, the KEGG analysis identified the signaling pathways involving these genes, including proteoglycans in cancer, the PI3K-Akt signaling pathway, focal adhesion, the MAPK signaling pathway, and the Rap1 signaling pathway (Figures 2(c) and 2(d)).

### 3.3. Consensus Clustering Classified Patients according to Prognostic mARGs and Relevant Analyses

**Figure 3:**
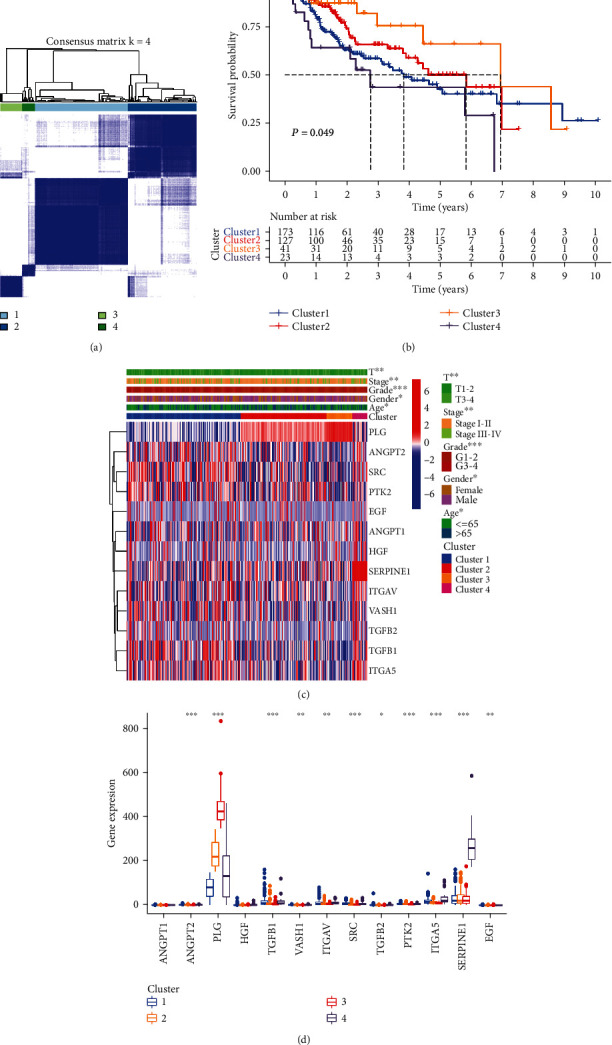
Consensus clustering for prognosis-related mARGs and related analyses. (a) Based on the consensus clustering matrix, patients with HCC were classified into four clusters. (b) Kaplan-Meier survival curves showed differences in prognosis between the four clusters. (c) Heat map and clinicopathologic characteristics among the four clusters. (d) Box plot of differential expression of prognostic-related mARGs in the four clusters. ^∗^*P* < 0.05, ^∗∗^*P* < 0.01, and ^∗∗∗^*P* < 0.001.

Based on the similarity of expression of prognosis-related mARGs, HCC patients were classified into different clusters using a consensus clustering method. *K* = 4 was found to have the best cluster stability for *K* = 2 to 9 (Figure 3(a)). The patients in these four clusters differed in prognosis, with cluster 3 showing the best prognosis and cluster 4, the worst prognosis (Figure 3(b)). The differential gene expression and clinical information between these four clusters were shown on the heat map in Figure 3(c). There were significant differences between the four clusters with respect to age, sex, grade, stage, and depth of infiltration. Overall, cluster 3 had a higher proportion of male, grades 1-2, stages I-II, and T1-2 patients than other clusters; cluster 1 had the highest proportion of patients aged ≤65; and cluster 4 had a lower proportion of males, stages I-II, and T1-2 patients than other clusters. PLG and SERPINE1 were significantly more expressed in clusters 3 and 4, respectively, than in other clusters (Figure 3(d)).

### 3.4. Construction of the Prognostic Signature and Subsequent Validation

**Figure 4:**
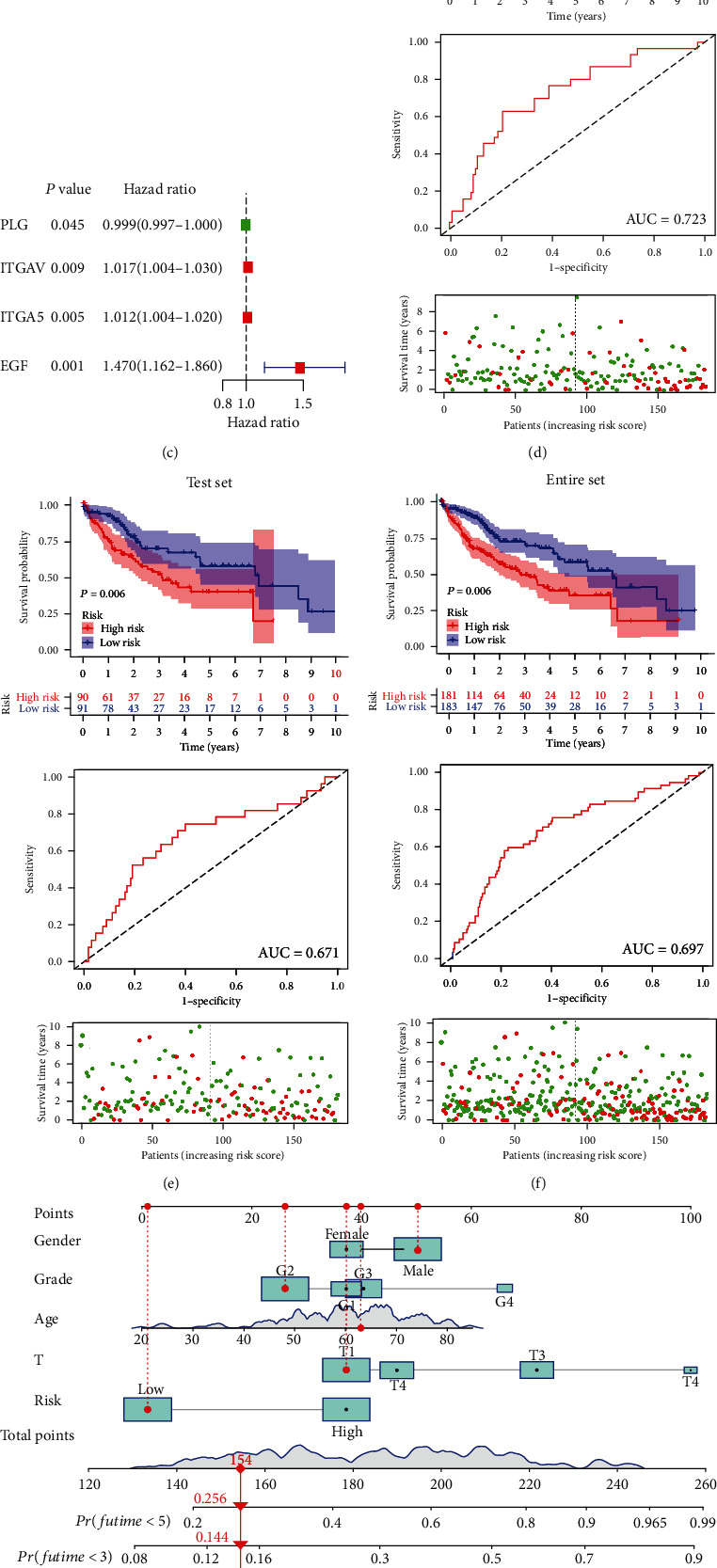
The mARG-signature construction and subsequent validation. (a and b) Four signature genes screened by LASSO regression analysis. (c) Forest plot of the LASSO regression analysis of the four signature genes. (d–f) Kaplan–Meier survival curves, ROC curves, and distribution of survival status and risk score of the training set, the test set, and the entire set. (g) Nomogram for forecasting the 1-, 3-, and 5-year overall survival of HCC patients. (h) The ROC curves of the nomogram indicate its good performance.

A further LASSO regression analysis was performed using 13 prognostic mARGs, and 4 mARGs (*PLG*, *ITGAV*, *ITGA5*, and *EGF*) were selected to construct the signature (Figures 4(a) and 4(b), Supplementary Table 4). For each sample, the risk score was calculated using the formula: Risk score = (0.010992486∗ITGAV) + (0.010221660∗ITGA5)–(0.000042854∗PLG) + (0.198147611∗EGF).  Figure 4(c) shows the univariate Cox analysis results for the mARGs in the signature. *ITGAV*, *ITGA5*, and *EGF* in the signature were risk factors for HCC patients, and *PLG* was a protective factor for HCC. Based on risk grouping and patient survival information, we performed a Kaplan–Meier survival analysis using training, test, and entire sets (Figures 4(d)–4(f)). The results indicated that the prognosis of the high-risk group was worse. The ROC curves for the training, testing, and entire sets used 1 year as the endpoint. The AUC was 0.723 for the training set, 0.671 for the test set, and 0.697 for the entire set, and the concordance index of the risk score was higher than that of other indicators (Supplementary Figure S2A). In addition, the univariate Cox analysis and the multivariate Cox analysis revealed a remarkable association between the risk score and the prognosis of HCC patients (Supplementary Figure S2B, C). Taken together, these results indicated that our signature was more effective in predicting prognosis compared to other clinical indicators. Subsequently, we developed a nomogram (Figure 4(g)) for overall survival prediction using clinical indicators and risk scores. The AUCs of the nomogram predicting overall survival at 1, 3, and 5 years were 0.729, 0.696, and 0.848, showing its good performance (Figure 4(h)). The Sankey diagram demonstrated the distribution of patients in four clusters, two risk groups, and clinical outcomes (Supplementary Figure S2D).

### 3.5. Signature-Related Gene Set Enrichment Analysis, Cancer Stemness, Clinical Characteristics, and Genetic Mutation Analyses

**Figure 5:**
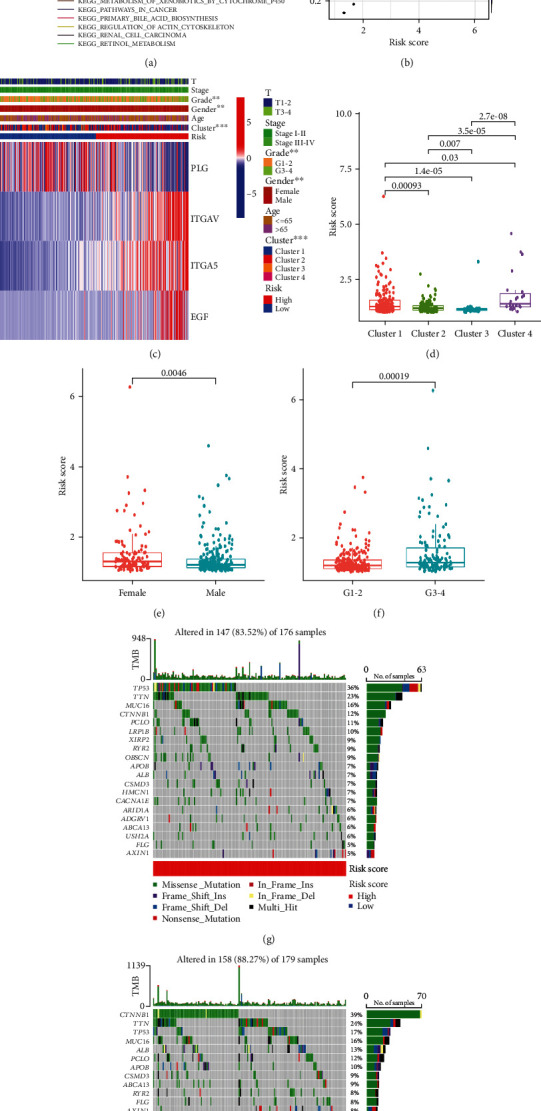
Signature-related functional enrichment, clinical characteristics, and gene mutation analysis. (a) The GSEA analysis result of the five most abundant KEGG pathways enriched in the high- and low-risk groups. (b) Analysis of the correlation between cancer cell stemness and risk scores. (c) Heat map of clinical characteristics between the two risk groups. Box plots of the differences in the risk score between (d) different groups, (e) different sexes, and (f) different grades. (g and h) Waterfall plot demonstrating the top 20 most mutated genes and mutation types in the two risk groups. ^∗∗^*P* < 0.01 and ^∗∗∗^*P* < 0.001.

To obtain additional information on the signaling pathways that differ in the two risk groups, we performed GSEA analysis and identified the five most enriched functional and signaling pathways in each of the two groups (Figure 5(a)). The high-risk group was enriched in endocytosis, cancer pathways, regulation of actin cytoskeleton, and ERBB signaling pathways, and the low-risk group was enriched primarily in bile acid biosynthesis, drug metabolism cytochrome P450, fatty acid metabolism, retinol metabolism, and metabolism of xenobiotics by cytochrome P450.

We also evaluated the potential relationship between signature and HCC stemness and between signature and oxidative stress. Overall, HCC stemness was negatively related to the risk score (*R* = −0.19, *P* = 4e − 04) (Figure 5(b)), indicating that patients with HCC with a lower risk score had more significant stem cell characteristics and lower levels of cell differentiation. In terms of oxidative stress levels, the expression of genes related to oxidative stress-related genes (i.e., *NFE2L2*, *NMOX1*, *TP53*, *NOS2*, and *NOS3*) were significantly higher in the high-risk group than in the low-risk group (Supplementary Figure S3).

We created a heat map and box diagrams to reveal the relationship among signature genes, clinical characteristics, and risk groups. As illustrated in the heat map (Figure 5(c)), in the high-risk group, *ITGAV*, *ITGA5*, and *EGF* were highly expressed, while *PLG* was lowly expressed. There were significant differences in sex and tumor grade between the two risk groups, with the ratio of female patients and the patient ratio of grades 3-4 significantly higher in the high-risk group. Risk scores were prominently different among the 4 clusters (Figure 5(d)), with the lowest risk score in cluster 3 and the highest risk score in cluster 4, which is consistent with the results of survival analysis of different clusters described above. When the patients were grouped by sex and grade, female and grades 3-4 patients had markedly higher risk scores (Figures 5(e) and 5(f)). Regarding the relationship between signature genes and clinical characteristics, PLG expression was significantly upregulated in male patients, aged over 65 years of age, T1-2, and grades 1-2; ITGA5 expression was significantly upregulated in female patients and grades 3-4; and EGF expression was significantly upregulated in grades 3-4 patients (Supplementary Figure S4A–D).

We then analyzed differences in somatic mutation characteristics between the two risk groups in HCC samples (Figures 5(g) and 5(h)). The mutation rates for *TTN*, *TP53*, *CTNNB1*, *MUC16*, and *PCLO* were greater than 10% in both groups. The mutation rates of *CTNNB1*, *TTN*, and *PCLO* were higher in the low-risk group, while *TP53* mutations were more common in the high-risk group.

### 3.6. Signature-Related Immune Infiltration and Functions, TME, and Immune Checkpoint Gene Analyses

**Figure 6:**
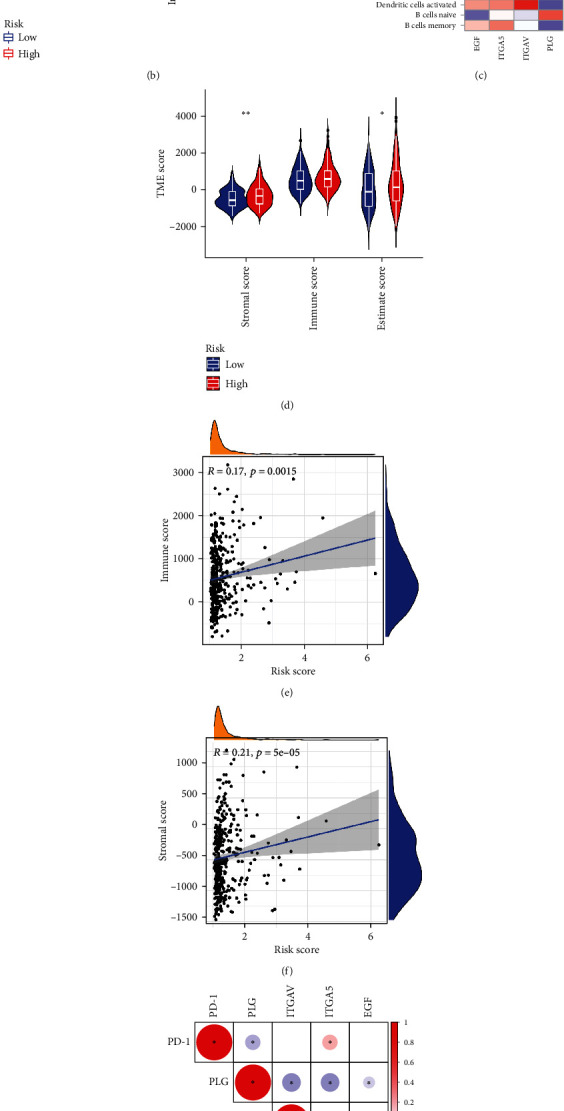
Signature-related immune landscape. (a) Violin plots show differences in the abundance of immune cells between the two risk groups. (b) Differences in immune-related functions between the two risk groups based on ssGSEA. (c) Correlation of 4 signature genes with immune cells. (d) Differences in the TME score between the two risk groups. (e) The relationship between the immune score and risk score. (f) The relationship between the stromal score and risk score. (g) Correlation among the three immune checkpoint genes and the 4 signature genes. Positive correlations are denoted in red, and negative correlations are denoted in blue; the presence of a statistically significant correlation between two RNAs is labeled with an asterisk. ^∗^*P* < 0.05, ^∗∗^*P* < 0.01, and ^∗∗∗^*P* < 0.001, ns: not significant.

Figure 6(a) reveals the abundance of 22 types of immune cells in the two risk groups, of which the abundance of plasma cells, CD8^+^ T cells, *γδ*T cells, and monocytes in the high-risk group appeared considerably lower, while the abundance of resting memory CD4^+^ T cells and M0 macrophages in the high-risk group was significantly higher. The risk score was positively associated with the abundance of activated dendritic cells, M0 macrophages, and neutrophils and negatively associated with the abundance of *γδ*T cells, plasma cells, and CD8^+^ T cells (Supplementary Figure S5A–F). Analysis of differences in scores for the 13 immune functions based on ssGSEA indicated that CCR, check point, MHC class I, and parainflammation scores were higher in the high-risk group, and the cytolytic activity score was higher in the low-risk group (Figure 6(b)).  Figure 6(c) shows the correlation of each signature gene with 22 immune cells: EGF expression was negatively related to naive B cell abundance; ITGA5 expression was positively related to M0 macrophage and neutrophil abundance and negatively related to monocyte, plasma cell, and *γδ*T cell abundance; ITGAV expression was positively correlated with activated dendritic cell, M0 macrophage, and resting NK cell abundance and negatively correlated with plasma cell, CD8^+^ T cell, T cells follicular helper, and *γδ*T cell abundance; and PLG expression was positively correlated with naive B cell abundance, while M1 macrophage abundance was positively correlated with memory B cell, activated dendritic cell, and M0 macrophage abundance. Furthermore, the stromal score and the estimate score in the high-risk group were markedly higher, indicating that the patients had lower tumor purity. Furthermore, the risk score was positively associated with the stromal score and the immune score (Figures 6(d)–6(f)).

We also examined the expression of the immune checkpoint gene expression. As shown in Supplementary Figure S5G, most immune checkpoint-related genes were expressed at higher levels in the high-risk group, leading to the hypothesis that patients in the high-risk group may experience more benefit from immune checkpoint inhibitors (ICIs). We analyzed the relationship between 3 immune checkpoint genes (*PD-1*, *PD-L1*, and *CTLA4*) and signature genes (Figure 6(g)). *PLG* was negatively related to all three immune checkpoint genes, *ITGA5* was positively related, *EGF* was positively related to *PD-L1*, and *ITGAV* was positively related to *CTLA4* and *PD-L1*.

These results revealed correlations between signature scores and the TME, immune-related pathways, and immune checkpoint genes.

### 3.7. Drug Correlation Analysis and Drug Screening

**Figure 7:**
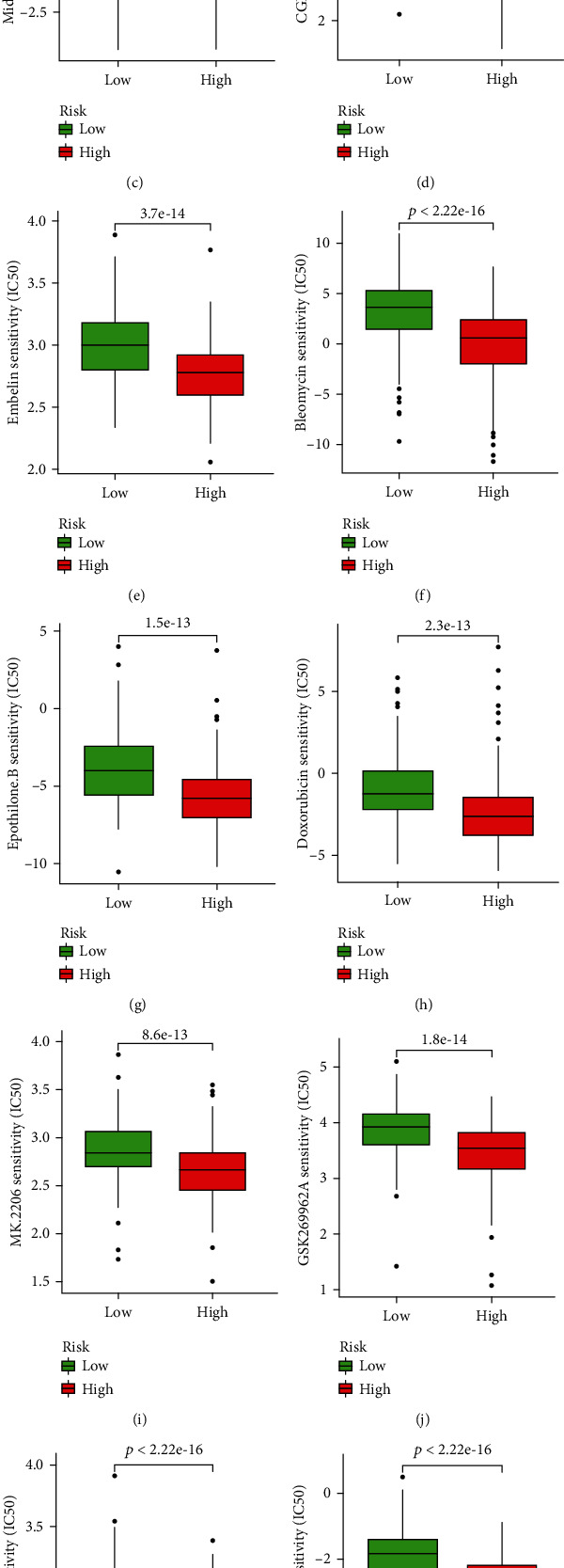
Antitumor drugs screening. (a) Correlation analysis of ITGAV expression and oxaliplatin drug sensitivity. (b) Correlation analysis of ITGAV expression and LDK-378 drug sensitivity. (c–l) 10 drugs with a lower IC50 in the high-risk group according to the GDSC database.

CellMiner was employed to identify drugs associated with signature genes. We found that the drug sensitivity of oxaliplatin and LDK-378 was negatively correlated with ITGAV expression (Figures 7(a) and 7(b)), suggesting that they may exert better antitumor effects in patients with lower ITGAV expression. Furthermore, Figures 7(c)–7(l) illustrated that embelin, bleomycin, epothilone B, midostaurin, CGP.082996, doxorubicin, MK.2206, GSK269962A, PF.562271, and gemcitabine had remarkably lower IC50 in the high-risk group, indicating that they were more effective at lower concentrations in the high-risk group. Furthermore, epothilone B may have the most potent antitumor efficacy in the high-risk group among the 10 drugs, as its IC50 was the lowest.

### 3.8. Detection and Validation of Signature Genes in Tissues

**Figure 8:**
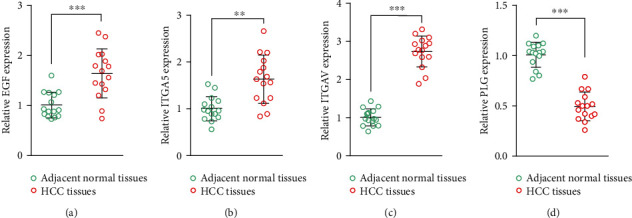
Expression of four signature genes in HCC and normal tissues (*n* = 15). (a) *EGF*. (b) *ITGA5*. (c) *ITGAV*. (d) *PLG*. ^∗∗^*P* < 0.01 and ^∗∗∗^*P* < 0.001.

Furthermore, we detected the expression levels of four signature genes in HCC and normal tissues by qRT-PCR. The qRT-PCR data from 15 patients showed a statistically significant increase in the expression of *EGF*, *ITGA5*, and *ITGAV* and a decrease in the expression of *PLG* in HCC tissues (Figures 8(a)–8(d)). Examining the signature genes at the tissue expression level verified the precision of our bioinformatic analysis.

## 4. Discussion

Angiogenesis is a vital aspect in cancer progression, as it provides the nutrients necessary for tumor growth. In HCC angiogenesis, many abnormally expressed genes participate in the regulation of this process. Increased secretion of *VEGFA* directly promotes angiogenesis [42]. Furthermore, the expression of HIF-1*α* is significantly upregulated in HCC, which in turn can transcriptionally upregulate the expression of *VEGFA*, *TGFB*, and *EPO* genes to stimulate angiogenesis [43]. The m^6^A modification, one of the most widespread RNA modifications, has been reported to regulate gene expression related to angiogenesis. In gastric cancer, *IGF2BP3*, an m^6^A reader, positively regulates *HIF-1α* gene and promotes angiogenesis through m^6^A regulation [44] and can also regulate the *VEGF* gene to facilitate angiogenesis in colon cancer [45]. Furthermore, *METTL3* could regulate the expression of the *TEK* and *VEGFA* genes to promote angiogenesis and exacerbate bladder cancer progression [46]. More importantly, previous studies have shown that angiogenic factors can interact with a variety of immune cells, such as tumor-associated macrophages, implying that angiogenesis is closely linked to tumor immunity [47]. Although the critical contribution of angiogenesis in HCC progression is evident and angiogenesis-related genes are widely modified by m^6^A in a variety of diseases, most existing studies have only investigated the mechanisms of regulation of ARG by a specific m^6^A regulator [27]. However, there is a lack of studies on mARGs as signature genes to predict prognosis, reveal TME characteristics, and screen for drugs in patients with HCC.

In this study, after differential expression analysis, coexpression analysis, and univariate Cox regression analysis, we obtained 13 prognostic mARGs, and then GO and KEGG analyses were performed. HCC patients were classified into four clusters according to the expression pattern of prognostic mARGs using an unsupervised consensus clustering approach. There were significant differences in clinical characteristics and prognosis between the 4 clusters, with cluster 4 having the worst prognosis and cluster 3 having a relatively good prognosis. Then, using LASSO regression analysis, we constructed a 4-gene signature that included *PLG*, *ITGAV*, *ITGA5*, and *EGF*. *PLG* was downregulated in HCC and was a protective factor for prognosis, while *ITGAV*, *ITGA5*, and *EGF* were upregulated in HCC and were considered risk factors for prognosis. In particular, previous studies revealed that some of these genes were strongly involved in HCC. *EGF* was reported to be highly expressed in HCC and enhanced the metastatic ability of HCC cells through the regulation of fibronectin [48]. *ITGAV* regulates the invasive ability of HCC cells [49], and *ITGA5* facilitated HCC progression and was related to worse OS [50].

As shown by the results of the ROC curves, the C-index, and the Cox regression analysis, our model was able to independently predict the prognosis of HCC patients, and its efficacy was superior to other conventional prediction methods. Based on the expression of the signature genes, the risk scores were calculated and used for the risk groups. In general, prognosis was significantly worse in the high-risk group, with a higher proportion of female and poorly classified patients. The GSEA results showed that the high-risk group was more enriched in cancer-related pathways, while the low-risk group was more enriched in metabolic synthesis-related pathways, which could partially explain the different prognosis of patients between the two groups.

The signature is also tightly related to oxidative stress and the immune landscape. In the high-risk group, the expression of genes associated with oxidative stress was significantly higher than in the low-risk group, suggesting that patients in the high-risk group may have higher overall levels of oxidative stress, which may be closely related to their poorer prognosis. In terms of immune infiltration, the high-risk group had more abundant resting memory CD4^+^ T cells and M0 macrophages, while the high-risk group had fewer plasma cells, CD8^+^ T cells, *γδ*T cells, and monocytes than the low-risk group. Among those immune cells with significantly changed content, plasma cells have been shown to have a positive or neutral effect on the prognosis of most cancers [51], and high expression of plasma cell signature genes in nonsmall cell lung cancer immunotherapy is associated with better prognosis [52]. A higher abundance of CD8^+^ T cells is associated with a better prognosis [53, 54], and therefore reduced levels of CD8^+^ T cells may promote tumor progression. *γδ*T cells can suppress tumors in direct or indirect ways [55]; for instance, IFN-*γ* and TNF-*α*, which inhibit tumor growth, are sourced from *γδ*T cells [56, 57]. Regarding immune functions, cytolytic activity and type II IFN enrichment decreased significantly in the high-risk group, while MHC class I and parainflammation enrichment increased significantly. Cytolytic activity is positively relevant to the prognosis in melanoma and HCC [58, 59], and IFN-*γ*, the sole member of the type II INF family [60], performs pivotal antitumor functions [61–63]. Phagocytosis of macrophages is crucially regulated by MHC class I, and tumors with high expression of MHC class I are more resistant to anti-CD47 antibody therapy [64]. Furthermore, the stromal score and the estimate score were higher in the high-risk group than in the low-risk group, and the risk score was positively correlated with the stromal score and the immune score, indicating that the high-risk group had a higher TME score and lower tumor purity. Collectively, the variation in the content of these immune cells and immune functions between the two groups suggests a connection between the risk score and the TME.

ICIs have been used increasingly in cancer treatment and have improved prognosis [65, 66]. The high expression of immune checkpoint genes means that targeted therapy with immune checkpoint may be beneficial. The prognosis for patients in the high-risk group is worse, but the expression of its immune checkpoint gene was higher. The therapeutic efficacy of existing *PD-1* and/or *CTLA4* inhibitors was not different between the two groups; however, numerous emerging clinical trials evaluating ICIs in HCC are underway (e.g., targeting TIGIT, CD80, and LAG-3) [67], painting a brighter prospect for the treatment of patients in high-risk groups. Furthermore, to suggest potential new strategies for the treatment of HCC, we conducted a drug correlation analysis between signature genes and drugs and then found that the drug sensitivity of oxaliplatin and LDK-378 was negatively correlated with ITGAV expression. Oxaliplatin is a standard treatment option for HCC [41], and LDK-378, a prospective inhibitor of anaplastic lymphoma kinase [68], was shown to promote apoptosis and suppress the proliferation of HCC cells [69]. The 10 anti-HCC drugs screened in the GDSC database, including embelin, bleomycin, epothilone B, midostaurin, CGP.082996, doxorubicin, MK.2206, GSK269962A, PF.562271, and gemcitabine, had lower IC50 in the high-risk group, demonstrating their potential superior performance against cancer in the high-risk group and offering the possibility of reducing the risk of progression in patients with HCC. Of these, epothilone B had the lowest IC50 value.

This study presented some limitations. Our data was obtained from a public database whose sample selection bias may affect the accuracy and generalizability of our prognostic signature. Therefore, further extensive validation may be desired to confirm the robustness of the signature. In future, retrospective studies of public data should be combined with prospective studies.

## 5. Conclusions

We investigated the interaction and prognostic value of mARGs in HCC and the association of these genes with the tumor microenvironment. Our results suggest that the signature constructed here is a promising instrument for forecasting the prognosis of HCC patients and has a unique role in revealing the tumor immune microenvironment and thus possibly providing prospective therapeutic targets for improving the effectiveness of immunotherapy in HCC.

## Figures and Tables

**Table 1:** Univariate Cox regression analysis screened 13 prognostic-related mARGs.

Gene	Univariate Cox	
	HR (95% CI)	*P* value
ANGPT1	1.407895 (1.08147-1.832847)	0.011024
ANGPT2	1.171274 (1.022637-1.341514)	0.022416
PLG	0.998565 (0.997166-0.999966)	0.044633
HGF	1.065979 (1.010572-1.124423)	0.01897
TGFB1	1.009251 (1.002462-1.016085)	0.007491
VASH1	1.338667 (1.086052-1.65004)	0.006264
ITGAV	1.017021 (1.004166-1.030041)	0.009308
SRC	1.046818 (1.017093-1.077412)	0.001851
TGFB2	1.041059 (1.007923-1.075286)	0.014764
PTK2	1.07161 (1.016875-1.129291)	0.009722
ITGA5	1.0117 (1.003588-1.019878)	0.004629
SERPINE1	1.001974 (1.000184-1.003768)	0.030664
EGF	1.470273 (1.162034-1.860275)	0.001323

## Data Availability

The raw data analyzed during the current study are available from the corresponding author on reasonable request.

## Supplementary Materials

Supplementary 1 Materials Supplementary Figure S1: the flowchart of this study.

Supplementary 2 Supplementary Figure S2: prognostic signature validation. (A) C-index of the risk score. (B and C) Univariate and multivariate analyses of the risk score and clinical characteristics. (D) Sankey diagram showed the distribution of HCC patients.

Supplementary 3 Supplementary Figure S3: expression levels of oxidative stress-related genes in high and low risk groups. ^∗^*P* < 0.05, ^∗∗^*P* < 0.01, and ^∗∗∗^*P* < 0.001.

Supplementary 4 Supplementary Figure S4: association between grouping of different clinical characteristics and the 4 signature genes expression. (A) Age. (B) Sex. (C) Grade. (D) T stage.

Supplementary 5 Supplementary Figure S5: signature-related immune landscape analyses. (A–F) The correlation among the risk score and the abundance immune cells. (G) Differential expression of immune checkpoint genes in the two risk groups.

Supplementary 6 Supplementary Table 1: the primer sequences used in this study. Supplementary Table 2: list of 45 differentially expressed ARGs. Supplementary Table 3: results of correlation analysis of angiogenesis-related genes and m^6^A regulator genes. Supplementary Table 4: results of LASSO regression analysis.
