# Supplementary figures and images for: m6A-Related Angiogenic Genes to Construct Prognostic Signature, Reveal Immune and Oxidative Stress Landscape, and Screen Drugs in Hepatocellular Carcinoma
Source: Oxid Med Cell Longev. 2022 Sep 30;2022:8301888. doi: 10.1155/2022/8301888 (PMC9554665; doi:10.1155/2022/8301888)

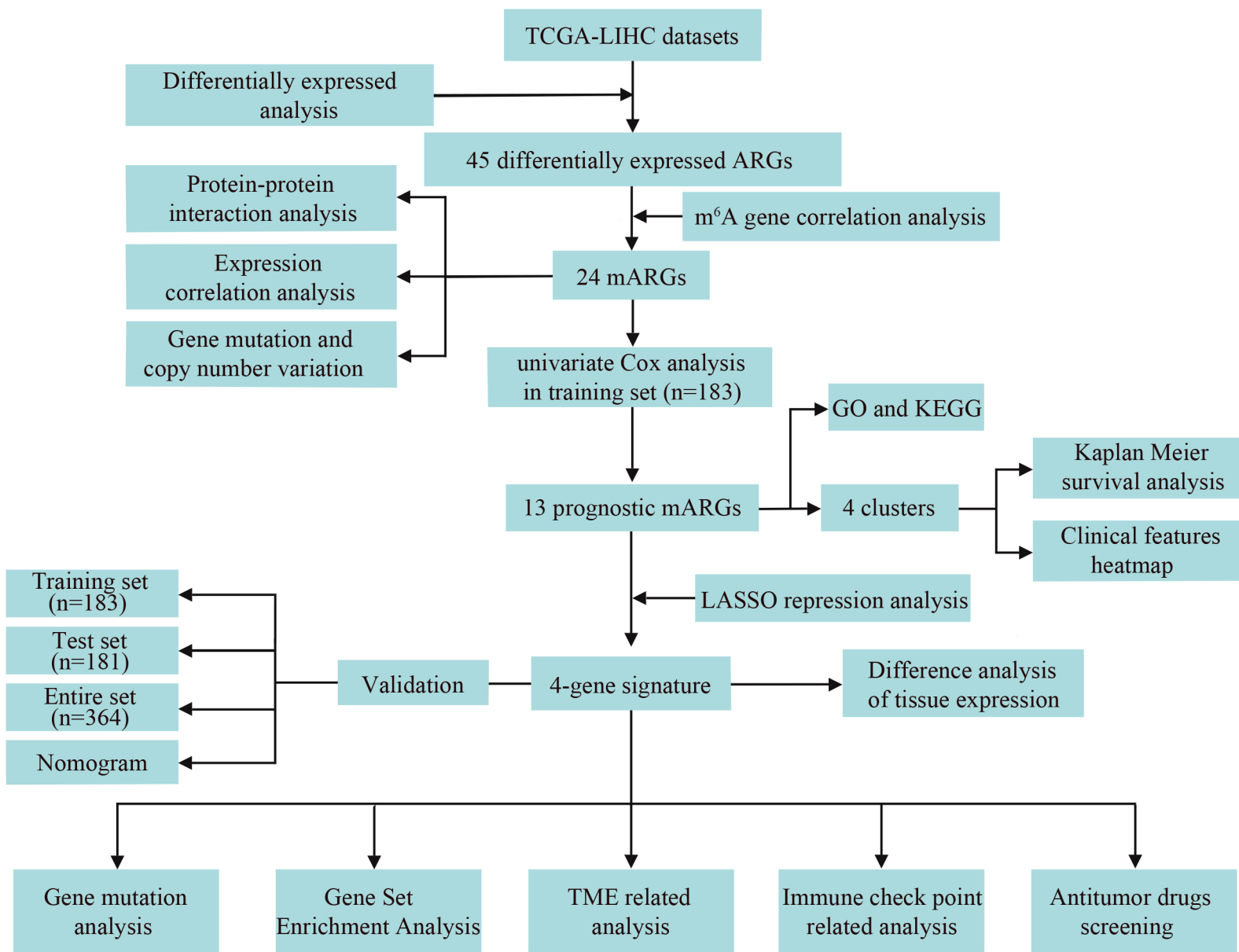

Supplement: Supplementary 1 — Materials Supplementary Figure S1: the flowchart of this study. [file 8301888.f1.pdf]

A

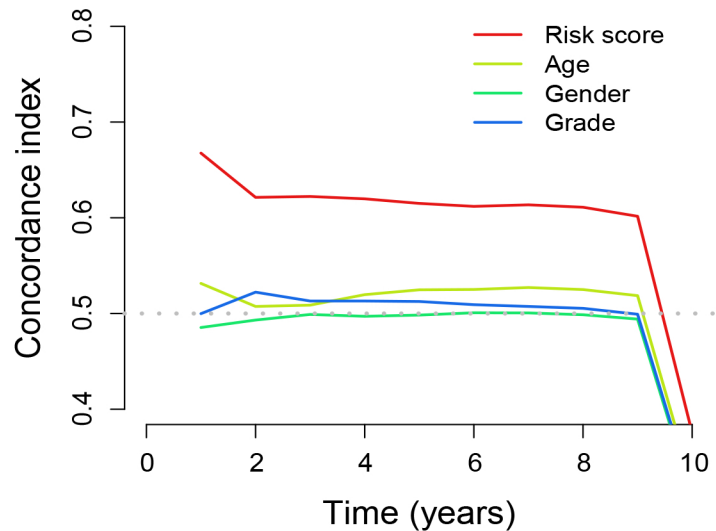

B

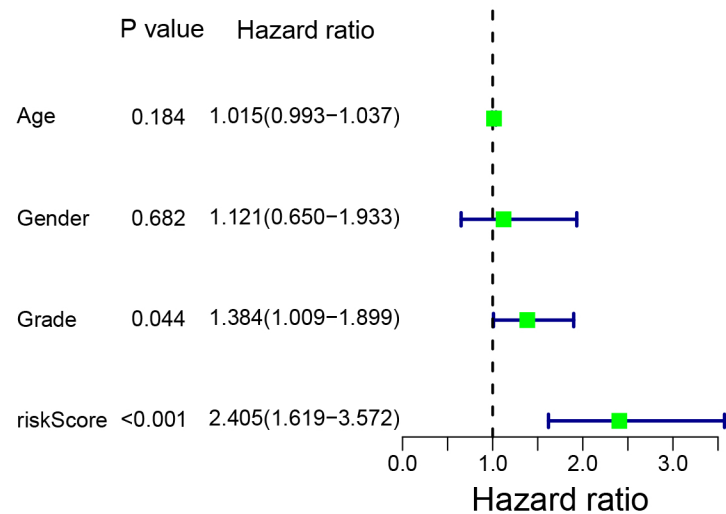

C

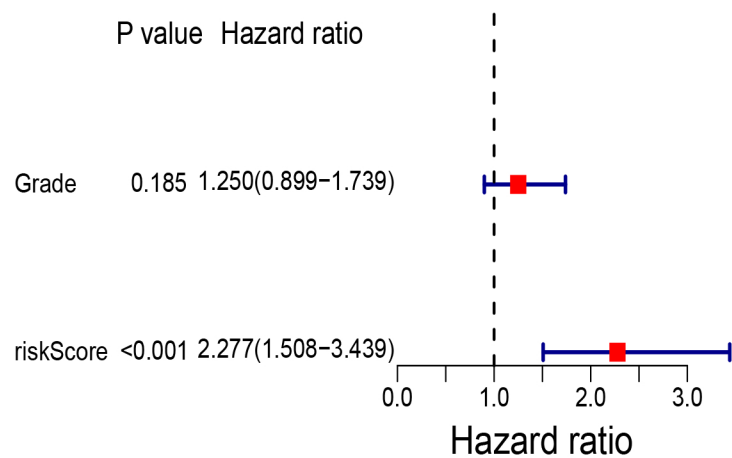

D

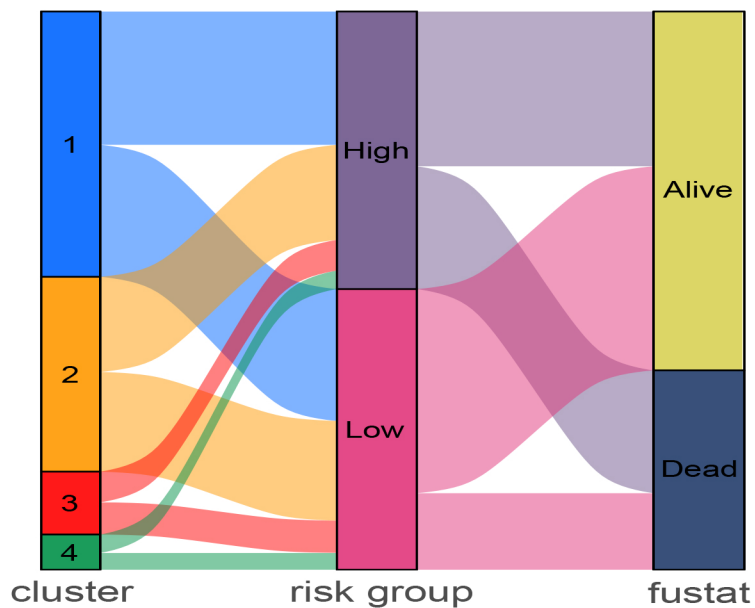

Supplement: Supplementary 2 — Supplementary Figure S2: prognostic signature validation. (A) C-index of the risk score. (B and C) Univariate and multivariate analyses of the risk score and clinical characteristics. (D) Sankey diagram showed the distribution of HCC patients. [file 8301888.f2.pdf]

Risk low high

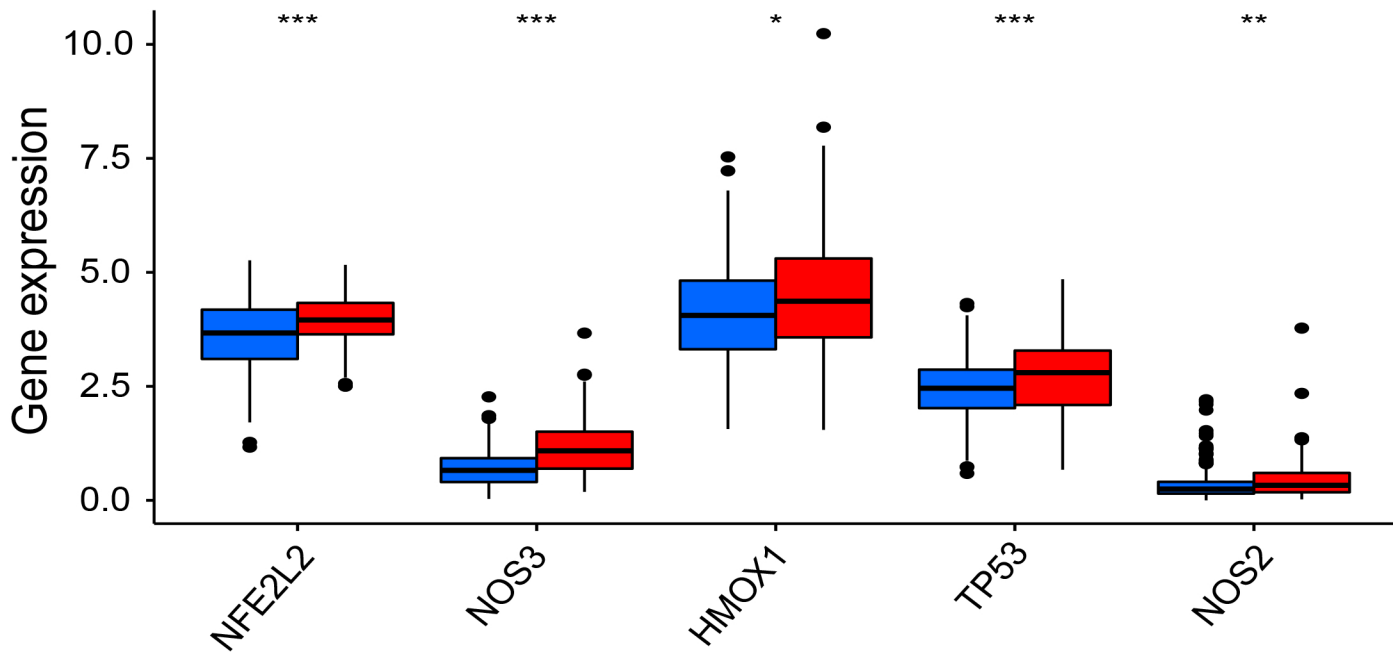

Supplement: Supplementary 3 — Supplementary Figure S3: expression levels of oxidative stress-related genes in high and low risk groups. ∗P < 0.05, ∗∗P < 0.01, and ∗∗∗P < 0.001. [file 8301888.f3.pdf]

**A**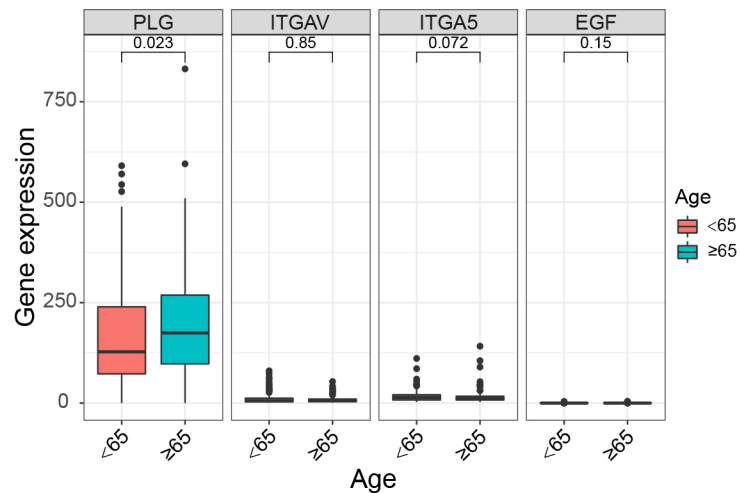**B**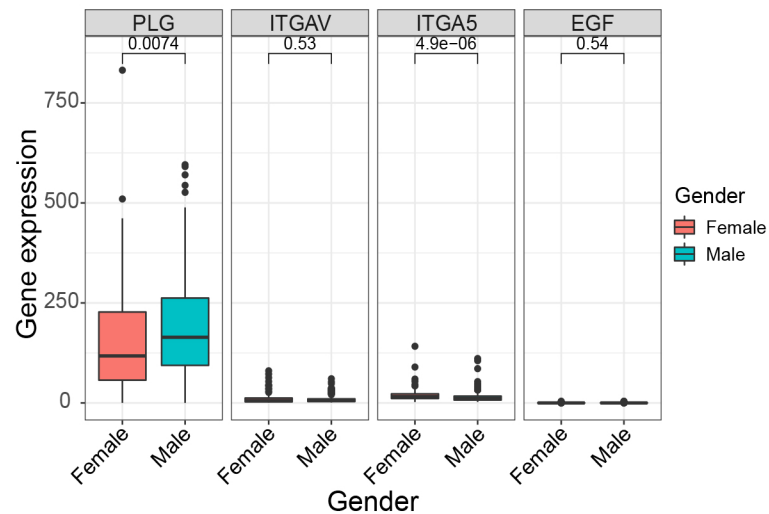**C**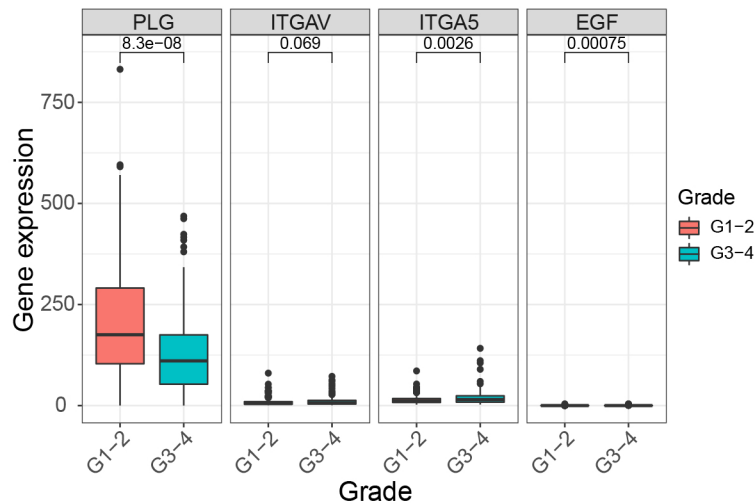**D**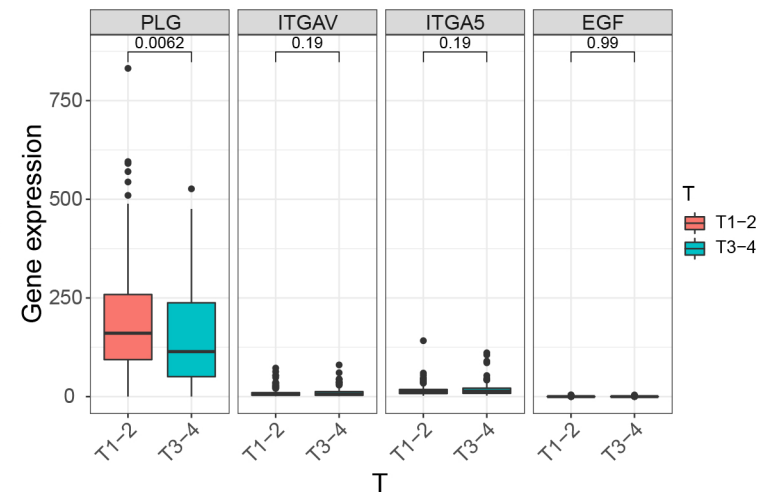

Supplement: Supplementary 4 — Supplementary Figure S4: association between grouping of different clinical characteristics and the 4 signature genes expression. (A) Age. (B) Sex. (C) Grade. (D) T stage. [file 8301888.f4.pdf]

**A**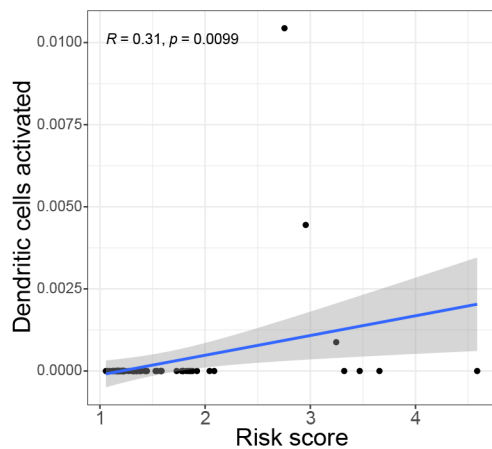**B**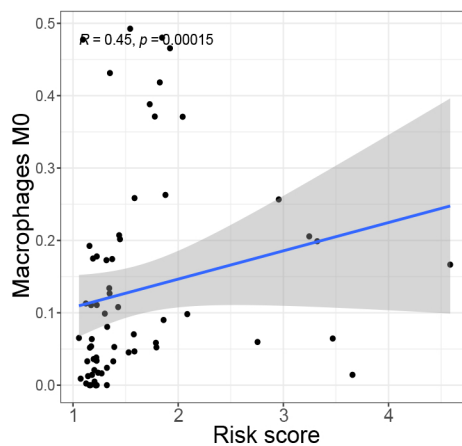**C**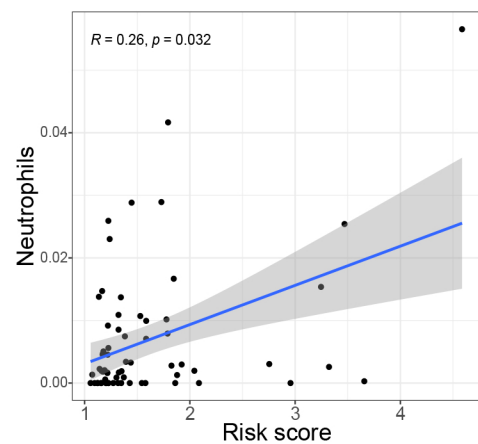**D**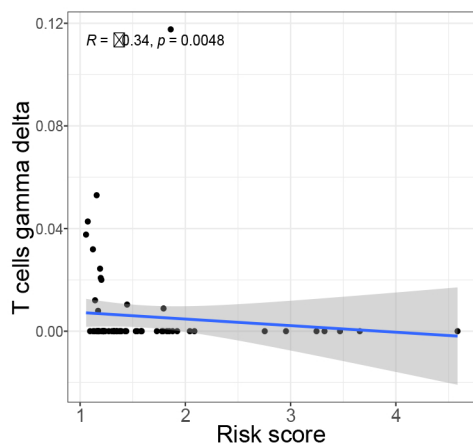**E**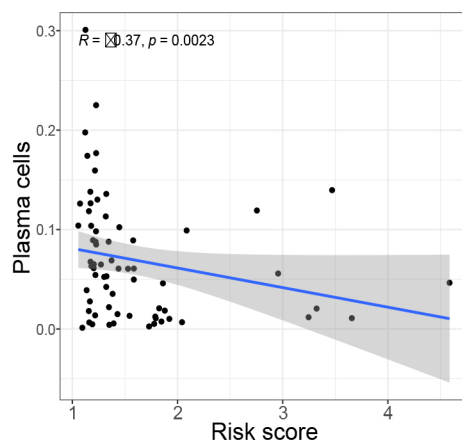**F**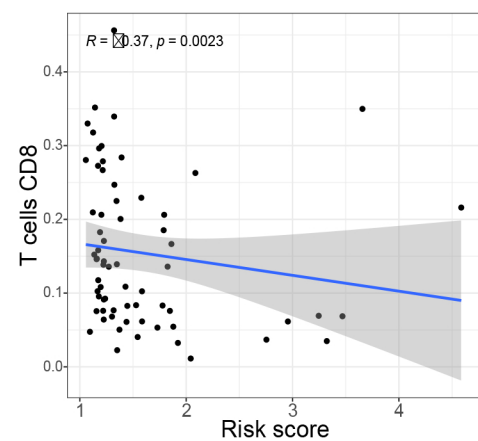**G**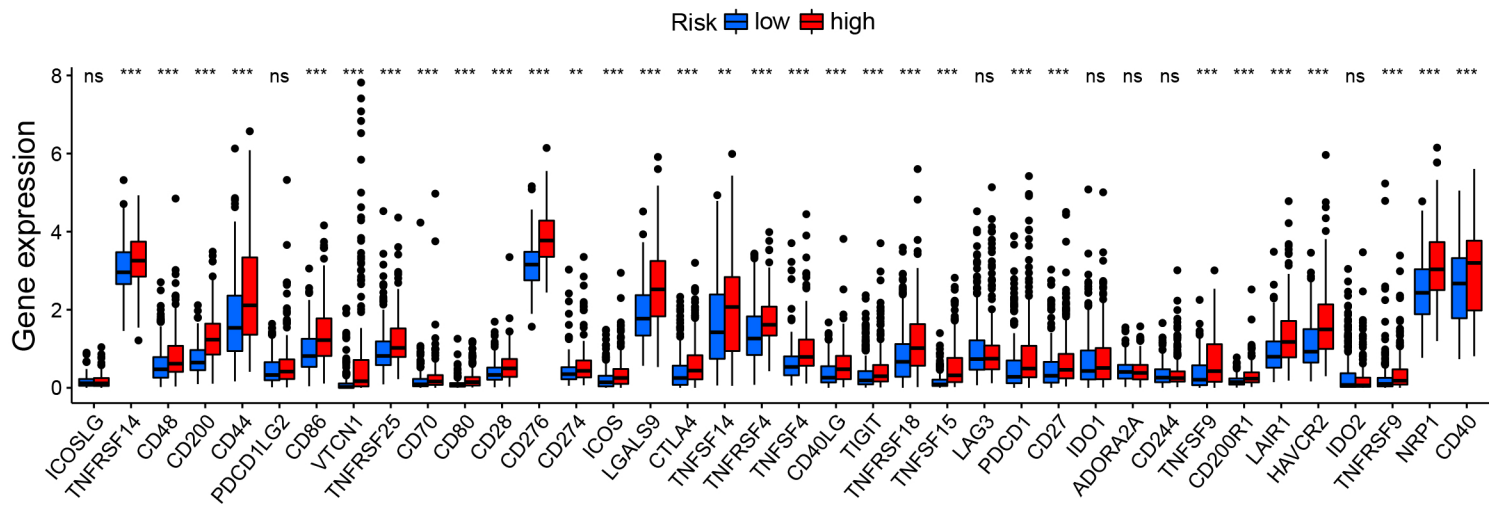

Supplement: Supplementary 5 — Supplementary Figure S5: signature-related immune landscape analyses. (A–F) The correlation among the risk score and the abundance immune cells. (G) Differential expression of immune checkpoint genes in the two risk groups. [file 8301888.f5.pdf]
