# Supplementary material for: m6A-Related Angiogenic Genes to Construct Prognostic Signature, Reveal Immune and Oxidative Stress Landscape, and Screen Drugs in Hepatocellular Carcinoma
Source: Oxid Med Cell Longev. 2022 Sep 30;2022:8301888. doi: 10.1155/2022/8301888 (PMC9554665; doi:10.1155/2022/8301888)
Supplement: Supplementary 6 — Supplementary Table 1: the primer sequences used in this study. Supplementary Table 2: list of 45 differentially expressed ARGs. Supplementary Table 3: results of correlation analysis of angiogenesis-related genes and m6A regulator genes. Supplementary Table 4: results of LASSO regression analysis. [file 8301888.f6.docx]

**Supplementary Materials**

**Supplementary Tables**

**Supplementary Table 1. The primer sequences used in this study.**

| Gene | Primer sequence (5′-3′) | |
| --- | --- | --- |
|  | Forward primer | Reverse primer |
| ITGAV | GACAGTCCTGCCGAGTA | CTGGGTGGTGTTTGCT |
| ITGA5 | GGTCGGGGGCTTCAACTTA | GAGCCGAGAGCCTTTGCTG |
| PLG | CAGGGGGCTTCACTGTTCAG | GCCATTATCACACATTGTTGCTC |
| EGF | TGCCAACTGGGGGTGCACAG | CTGCCCGTGGCCAGCGTGGC |
| GAPDH | CGGATTTGGTCGTATTGGG | CGCTCCTGGAAGATGGTGAT |

**Supplementary Table 2. List of 45 differentially expressed ARGs.**

| FLT1 | VEGFB | IL6 | EFNB2 | TYMP | VWF |
| --- | --- | --- | --- | --- | --- |
| ANGPT1 | IL1B | TGFB1 | VASH2 | ACVRL1 | CD36 |
| THBS1 | CCN1 | VASH1 | PLAU | SRC | PGF |
| ANGPT2 | PDGFB | VEGFD | ITGA5 | HGF | EPHA2 |
| ADGRB3 | CXCL12 | ITGAV | NOS2 | SPP1 | ID1 |
| ADGRB2 | TGFB2 | DLL4 | SERPINE1 | PTGS2 | MMP14 |
| PLG | ACE | PDGFRB | EGF | GDF2 | ECM1 |
| MMP9 | PTK2 | PECAM1 |  |  |  |

**Supplementary Table 3. Results of correlation analysis of angiogenesis-related genes and m^6^A regulator genes.**

| Angiogenesis | m^6^A regulator | cor | P value | regulation |
| --- | --- | --- | --- | --- |
| ACE | YTHDC1 | 0.347845 | 4.46E-12 | positive |
| ANGPT1 | WTAP | 0.341873 | 1.08E-11 | positive |
| ANGPT1 | YTHDF2 | 0.313805 | 5.44E-10 | positive |
| ANGPT1 | FTO | 0.355071 | 1.49E-12 | positive |
| ANGPT2 | YTHDC1 | 0.322542 | 1.68E-10 | positive |
| ANGPT2 | IGFBP3 | 0.342795 | 9.44E-12 | positive |
| CCN1 | YTHDC1 | 0.361339 | 5.61E-13 | positive |
| CCN1 | FTO | 0.34322 | 8.87E-12 | positive |
| CD36 | FTO | 0.349999 | 3.22E-12 | positive |
| EFNB2 | YTHDC1 | 0.330123 | 5.86E-11 | positive |
| EGF | RBM15 | 0.31593 | 4.10E-10 | positive |
| EGF | YTHDF2 | 0.348798 | 3.86E-12 | positive |
| EPHA2 | YTHDC1 | 0.306047 | 1.50E-09 | positive |
| FLT1 | YTHDC1 | 0.355933 | 1.30E-12 | positive |
| FLT1 | FTO | 0.384213 | 1.33E-14 | positive |
| HGF | IGFBP3 | 0.378864 | 3.27E-14 | positive |
| IL1B | WTAP | 0.368493 | 1.80E-13 | positive |
| IL1B | YTHDF2 | 0.32563 | 1.10E-10 | positive |
| IL1B | HNRNPC | 0.32463 | 1.26E-10 | positive |
| IL1B | HNRNPA2B1 | 0.304036 | 1.94E-09 | positive |
| ITGA5 | WTAP | 0.38108 | 2.26E-14 | positive |
| ITGA5 | YTHDC1 | 0.417719 | 3.17E-17 | positive |
| ITGA5 | YTHDF1 | 0.429635 | 3.12E-18 | positive |
| ITGA5 | YTHDF2 | 0.321293 | 1.99E-10 | positive |
| ITGA5 | HNRNPC | 0.367131 | 2.24E-13 | positive |
| ITGA5 | LRPPRC | 0.313385 | 5.75E-10 | positive |
| ITGA5 | HNRNPA2B1 | 0.40149 | 6.43E-16 | positive |
| ITGA5 | RBMX | 0.390279 | 4.68E-15 | positive |
| ITGAV | METTL16 | 0.317625 | 3.27E-10 | positive |
| ITGAV | WTAP | 0.385213 | 1.12E-14 | positive |
| ITGAV | RBM15 | 0.354154 | 1.71E-12 | positive |
| ITGAV | YTHDC1 | 0.568852 | 1.90E-33 | positive |
| ITGAV | YTHDC2 | 0.400468 | 7.72E-16 | positive |
| ITGAV | YTHDF1 | 0.31761 | 3.27E-10 | positive |
| ITGAV | YTHDF2 | 0.461593 | 3.91E-21 | positive |
| ITGAV | HNRNPC | 0.393746 | 2.55E-15 | positive |
| ITGAV | LRPPRC | 0.391193 | 3.99E-15 | positive |
| ITGAV | HNRNPA2B1 | 0.418758 | 2.60E-17 | positive |
| ITGAV | RBMX | 0.443316 | 1.95E-19 | positive |
| ITGAV | FTO | 0.375567 | 5.66E-14 | positive |
| MMP14 | METTL16 | 0.366424 | 2.50E-13 | positive |
| PDGFB | YTHDC1 | 0.400378 | 7.85E-16 | positive |
| PDGFRB | YTHDC1 | 0.36099 | 5.93E-13 | positive |
| PLG | METTL16 | -0.39845 | 1.11E-15 | negative |
| PLG | RBM15B | -0.36647 | 2.49E-13 | negative |
| PLG | YTHDF1 | -0.38218 | 1.87E-14 | negative |
| PLG | HNRNPC | -0.36763 | 2.06E-13 | negative |
| PLG | RBMX | -0.36639 | 2.52E-13 | negative |
| PTK2 | METTL3 | 0.404038 | 4.05E-16 | positive |
| PTK2 | VIRMA | 0.801072 | 6.28E-85 | positive |
| PTK2 | RBM15B | 0.410636 | 1.20E-16 | positive |
| PTK2 | YTHDC1 | 0.328989 | 6.87E-11 | positive |
| PTK2 | YTHDF1 | 0.326756 | 9.38E-11 | positive |
| PTK2 | YTHDF2 | 0.307437 | 1.25E-09 | positive |
| PTK2 | YTHDF3 | 0.588489 | 3.18E-36 | positive |
| PTK2 | HNRNPC | 0.378201 | 3.66E-14 | positive |
| PTK2 | LRPPRC | 0.54788 | 1.10E-30 | positive |
| PTK2 | HNRNPA2B1 | 0.356791 | 1.14E-12 | positive |
| PTK2 | RBMX | 0.409899 | 1.38E-16 | positive |
| SERPINE1 | IGFBP3 | 0.368331 | 1.84E-13 | positive |
| SRC | METTL3 | 0.37916 | 3.11E-14 | positive |
| SRC | METTL16 | 0.388217 | 6.69E-15 | positive |
| SRC | WTAP | 0.348327 | 4.15E-12 | positive |
| SRC | VIRMA | 0.430977 | 2.39E-18 | positive |
| SRC | RBM15 | 0.327003 | 9.06E-11 | positive |
| SRC | RBM15B | 0.481117 | 4.60E-23 | positive |
| SRC | YTHDC1 | 0.465404 | 1.68E-21 | positive |
| SRC | YTHDF1 | 0.553393 | 2.16E-31 | positive |
| SRC | YTHDF2 | 0.331896 | 4.56E-11 | positive |
| SRC | HNRNPC | 0.470497 | 5.34E-22 | positive |
| SRC | LRPPRC | 0.410931 | 1.14E-16 | positive |
| SRC | HNRNPA2B1 | 0.434964 | 1.08E-18 | positive |
| SRC | RBMX | 0.454086 | 2.00E-20 | positive |
| SRC | ALKBH5 | 0.303951 | 1.96E-09 | positive |
| TGFB1 | METTL16 | 0.378763 | 3.33E-14 | positive |
| TGFB1 | YTHDC1 | 0.30579 | 1.55E-09 | positive |
| TGFB1 | HNRNPC | 0.311169 | 7.70E-10 | positive |
| TGFB1 | RBMX | 0.365757 | 2.79E-13 | positive |
| TGFB2 | YTHDC1 | 0.387167 | 8.01E-15 | positive |
| TGFB2 | RBMX | 0.320173 | 2.32E-10 | positive |
| VASH1 | METTL3 | 0.32097 | 2.08E-10 | positive |
| VASH1 | METTL16 | 0.367172 | 2.22E-13 | positive |
| VASH1 | WTAP | 0.308481 | 1.09E-09 | positive |
| VASH1 | RBM15B | 0.332519 | 4.17E-11 | positive |
| VASH1 | YTHDC1 | 0.504198 | 1.65E-25 | positive |
| VASH1 | YTHDC2 | 0.345885 | 5.98E-12 | positive |
| VASH1 | YTHDF1 | 0.302899 | 2.24E-09 | positive |
| VASH1 | HNRNPC | 0.348047 | 4.33E-12 | positive |
| VASH1 | RBMX | 0.375833 | 5.42E-14 | positive |
| VASH1 | FTO | 0.326795 | 9.33E-11 | positive |
| VEGFB | METTL16 | 0.316818 | 3.64E-10 | positive |
| VEGFB | RBM15B | 0.317148 | 3.48E-10 | positive |
| VEGFB | YTHDF1 | 0.343938 | 7.98E-12 | positive |

**Supplementary Table 4. Results of LASSO regression analysis**

| Gene | Coefficient |
| --- | --- |
| PLG | -0.000042854 |
| ITGAV | 0.010992486 |
| ITGA5 | 0.010221660 |
| EGF | 0.198147611 |

**Supplementary Figure Legends**

**Supplementary Figure S1. The flowchart of this study.**

**Supplementary Figure S2. Prognostic signature validation. (A)** C-index of the risk score. **(B, C)** Univariate and multivariate analyses of the risk score and clinical characteristics. **(D)** Sankey diagram showed the distribution of HCC patients.

**Supplementary Figure S3. Expression levels of oxidative stress-related genes in high and low risk groups.** *P < 0.05, **P < 0.01, ***P < 0.001.

**Supplementary Figure S4. Association between grouping of different clinical characteristics and the 4 signature genes expression.** **(A)** Age. **(B)** Sex. **(C)** Grade. **(D)** T stage.

**Supplementary Figure S5.** **Signature-related immune landscape analyses. (A-F)** The correlation among the risk score and the abundance immune cells. **(G)** Differential expression of immune checkpoint genes in the two risk groups.
